# A public repository for mass spectrometry imaging data

**DOI:** 10.1007/s00216-014-8357-8

**Published:** 2014-12-27

**Authors:** Andreas Römpp, Rui Wang, Juan Pablo Albar, Andrea Urbani, Henning Hermjakob, Bernhard Spengler, Juan Antonio Vizcaíno

**Affiliations:** 1 Institute of Inorganic and Analytical Chemistry, Justus Liebig University, Schubertstrasse 60, 35392 Giessen, Germany; 2 European Molecular Biology Laboratory, European Bioinformatics Institute, Wellcome Trust Genome Campus, Hinxton, Cambridge, CB10 1SD UK; 3 Centro Nacional de Biotecnologia/CSIC, UAM Campus Cantoblanco, Darwin 3, 28049 Madrid, Spain; 4 Department of Experimental Medicine and Surgery, University of Rome “Tor Vergata”, 00133 Rome, Italy; 5 IRCCS – Fondazione Santa Lucia, Via del Fosso di Fiorano 65, 00143 Rome, Italy

## Introduction

Mass spectrometry (MS) imaging is a very active field of research, and has seen impressive progress in recent years [1, 2]. The number of groups that are working on this topic is constantly increasing. However, the field is still very heterogeneous in terms of applied instrumentation and data processing methods. In addition, complex datasets are reduced to a set of two-dimensional “images,” which inevitably results in information loss. This simplified graphical representation also strongly depends on processing options such as color scale, intensity normalization, and spatial interpolation. Consequently, experimental data are presented in very diverse ways, and published results can therefore be difficult to evaluate and compare. With a growing number of published studies, the issue of standardization and quality control of MS imaging data is becoming more important. This is a natural process for any new field that is maturing. The MS-based proteomics community has been facing similar issues in the last decade, and this discipline is therefore discussed as a “role model” herein. Since its inception in 2002, the Proteomics Standards Initiative (http://www.psidev.info) has driven the development of a number of minimum reporting guidelines (called “minimum information about a proteomics experiment” documents) [3] and several standard data formats for the different data types relevant in proteomics. For example, for raw and processed MS data, the data standard is called mzML [4].

In addition, several data repositories were established about 10 years ago to address the demand for storage and availability of MS data in the public domain [5–9]. A big step forward in this area has been the establishment of the ProteomeXchange (PX; http://www.proteomexchange.org/) consortium [10], led by the PRIDE [9] and PeptideAtlas [8] resources. The overall aim of PX is to provide a common framework and infrastructure for the cooperation of proteomics resources by defining and implementing consistent, harmonized, user-friendly data deposition and exchange procedures among the members. Thanks to the guidelines promoted by several scientific journals and funding agencies, and the general perception that sharing data is good scientific practice, the culture in the proteomics community has evolved toward data deposition as part of the publication process.

In analogy to these activities in the MS proteomics field, similar mechanisms have been discussed and to some extent already implemented in the MS imaging community in recent years. A common data format for MS imaging—imzML—has been established [11]. This format is being used more and more, and the number of available tools is constantly growing (see http://www.imzml.org for more details). Reporting guidelines have been discussed for several years, and a first suggestion of those is included in this topical collection [12].

Nevertheless, owing to the lack of suitable resources, a missing element so far has been the possibility to make MS imaging datasets available in the public domain. Earlier attempts to develop a data repository were abandoned mainly because of the large size of MS imaging datasets. However, nowadays very large datasets (i.e., file size on the order of a few terabytes) can also be generated in MS-based proteomic and metabolomic studies, and can be submitted to established repositories.

From a purely technical point of view, the infrastructure available in existing MS repositories is also suited for MS imaging data. Therefore, the missing step is to define and adopt a submission procedure in order to be compatible with MS-imaging-specific parameters. Here we describe the newly implemented way of submitting MS imaging data to PX via the PRIDE database. We also describe how to retrieve these data and to reproduce the MS images.

## Experimental procedures/submission process

### Procedure for submitting datasets

For MS proteomics, there are two different PRIDE/PX submission modes: “complete” and “partial.” For both types of submission, a set of common metadata (agreed by all PX partners) and MS raw data are mandatory for each dataset. The difference is in the way the processed results are provided. After a “complete” submission has been performed it is possible for the repository to directly connect the processed peptide/protein identification results with the mass spectra. This can be achieved if the processed results are available in an open data format supported by the repository. The alternative is to perform a “partial” submission, and in this case, the connection between the spectra and the identification results cannot be done in a straightforward way. For “partial” submissions, the processed results are not available in a format supported by the repository. Instead, the corresponding analysis software’s output files (in heterogeneous formats) are made available for download [13].

The submission procedure has been adapted for MS imaging data using the “partial” submission mechanism. The PX Submission Tool [10] is the main tool used to perform the submissions. It is developed in Java and provides a user-friendly graphical user interface for performing the actual data submission, through a series of stages: (1) select all the files needed for the submission; (2) interactively group related files; (3) ensure the minimum level of metadata annotation; and (4) transfer the files via Aspera (http://asperasoft.com/) or FTP. Aspera can perform much faster transfers than FTP, resulting in a very convenient way of transferring potentially very large datasets.

In addition to the PX Submission Tool, datasets containing a high number of files can also be submitted using a command-line-based alternative [13].

An exemplary dataset has been used to demonstrate the submission process. The data describe a matrix-assisted laser desorption ionization imaging experiment of mouse urinary bladder tissue sections acquired with high mass accuracy at a pixel size of 10 μm which was reported previously [14]. Experimental details are described in the electronic supplementary material (the results published originally are shown in Fig. 2, panels A and B). The dataset was deposited in PRIDE/PX (accession number PXD001283).

The submission process is described below; screenshots of all submission steps are provided in Figs. S1–S10. For a detailed description of the submission process, a tutorial for MS proteomics data is available [13]. Here, we will mainly focus on the changes made to accommodate the MS imaging datasets.

Before starting, users must first register at PRIDE (http://www.ebi.ac.uk/pride/archive/register). The default assumption is that all of the files belonging to one study or manuscript will be uploaded at once and handled as a unit, although there is some flexibility in the process. After the PX Submission Tool has been launched, in step 1 the type of submission must be indicated. In this case, “partial” must always be chosen.

**Fig. 1:**
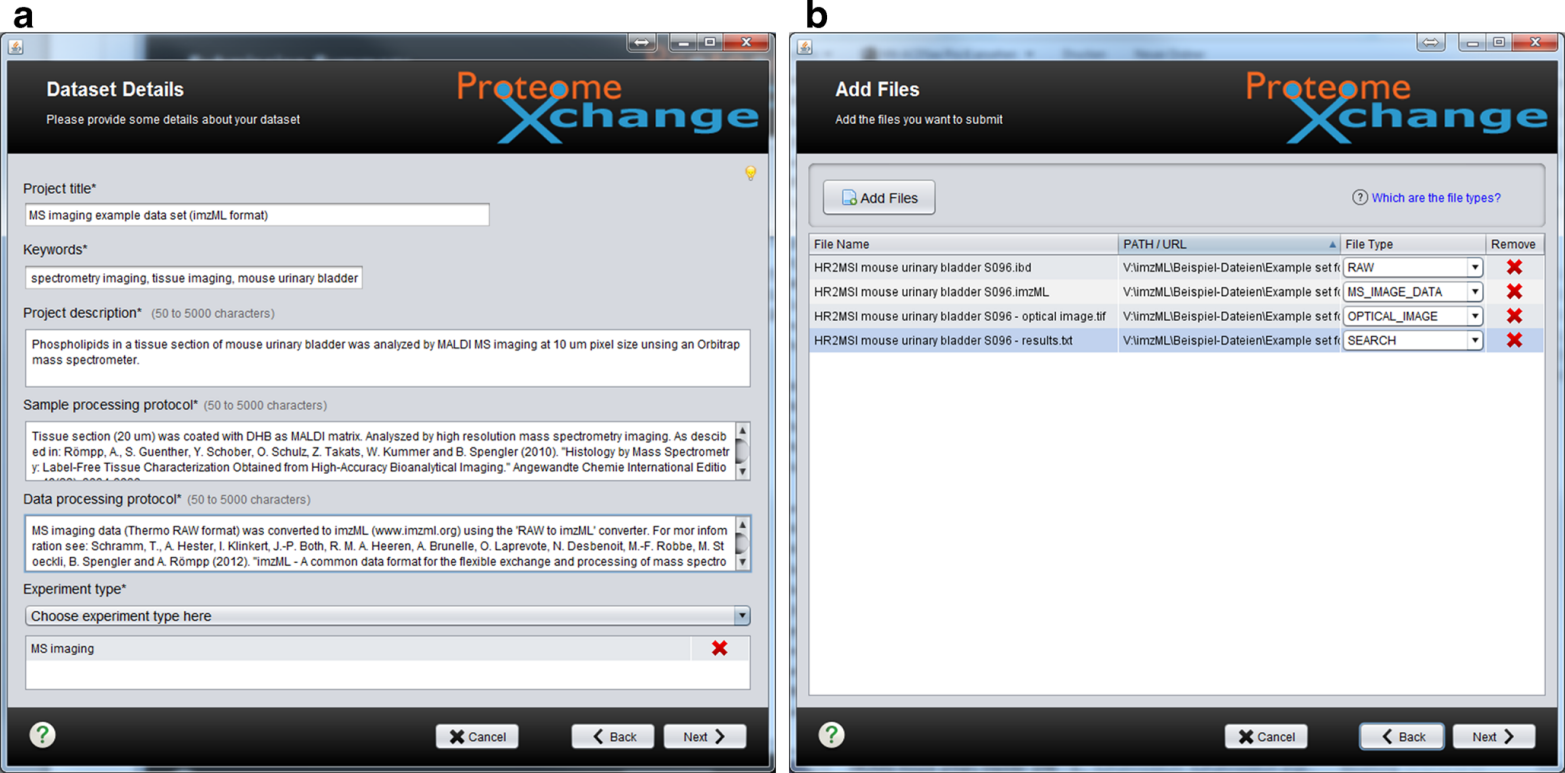
Screenshots of the ProteomeXchange submission tool: **a** step 2 “Dataset Details” and **b** step 3 “Add Files”

In step 2, metadata are provided to describe the overall study, such as title, description, sample processing and data processing protocols, keywords, and experiment type (Fig. 1a). Most of them can be provided as simple text. A new term called “MS imaging” (MS: 1002521) has been created in the PSI-MS controlled vocabulary [15] to include this new experiment type in the submission process. The sample processing protocol should include all details on sample preparation, data acquisition, and instrumentation used. A crucial value that needs to be provided in any case is the pixel size that was used to acquire the reported MS image. A general suggestion for parameters that should be reported for MS imaging experiments has been proposed recently [12]. The data processing protocol is of particular importance for reproducing the MS images shown in the publication. A critical parameter is the bin size of the MS images, i.e., the mass range that was used to generate the image (e.g., ±0.1 Da of the theoretical mass of the imaged compound). Other important details are the information about (pixelwise) intensity normalization as well as the type and level of spatial interpolation (and color map applied).

Actual data selection is performed in step 3 (“Add Files”) (Fig. 1b). These files include mass spectral data (labeled as “RAW”), metadata information about the images (labeled as “MS_IMAGE_DATA”), and an optical image as a reference (labeled as “OPTICAL_IMAGE”). The new file tags “MS_IMAGE_DATA” and “OPTICAL_IMAGE” have been incorporated to support MS imaging data. The files submitted for the example dataset are summarized in Table 1. The main specific points to consider for this step are as follows:

**Table 1:** Submitted data files in the example dataset (accession number PXD001283)

File name	File type	Comment
HR2MSI mouse urinary bladder S096.ibd	RAW	Binary part of imzML data
HR2MSI mouse urinary bladder S096.imzml	MS_IMAGE_DATA	XML part of imzML data
HR2MSI mouse urinary bladder S096 - optical image.tiff	OPTICAL_IMAGE	Optical image of measured sample (tissue section)
HR2MSI mouse urinary bladder S096 - results.txt	SEARCH	Information on MS images which have been presented in the corresponding manuscript (e.g., *m*/*z* values)

*MS* mass spectrometry

1. It is mandatory to provide the MS raw data (labeled as “RAW”). It is recommended to submit MS imaging data in imzML format as it offers the most flexible options for viewing, but proprietary data formats are also accepted. The default submission process has been modified, and it now includes the possibility to submit two different mass-spectral-related files for one dataset, as required for several MS imaging data formats (e.g., imzML and Analyze). The mass spectral data file (*.ibd file for imzML or *.img file in Analyze format) must be labeled as “RAW.” The file that contains metadata (such as pixel dimensions and additional information) must be labeled as “MS_IMAGE_DATA” (e.g., *.imzml file for imzML or *.hdr file for Analyze). If an “ibd” file (imzML format) is submitted as “RAW,” an “MS_IMAGE_DATA” file (*.imzml) is mandatory. However, in the case of “RAW” proprietary formats that consist of only one file, an “MS_IMAGE_DATA” file is not required.
2. In addition, PRIDE requires a mandatory “SEARCH” file for “partial” submissions, which corresponds to the processed results. This file typically contains a list of identified peptides and proteins, and related metadata. There is currently no strict definition for the format of this mandatory file, but it should contain a list of *m*/*z* values, names of (tentatively) identified compounds, and additional information that was used to generate the MS images in the published work. The “SEARCH” file for the example dataset is given in the electronic supplementary material. These data should be available from the manuscript, but including them in a concise way in the data submission facilitates the reproduction of the published MS images.
  We acknowledge that the file tag “SEARCH” might not be the best option to describe these data, but this file is still mandatory for consistency with the overall PX submission framework. Another more specific file tag might be added in the future if this turns out to be necessary.
3. Since MS imaging data contain spatial information, the data submission also supports the inclusion of an optical image (“OPTICAL_IMAGE”) of the measured sample, which can allow validation and/or interpretation. The “OPTICAL_IMAGE” file could contain a photograph of the imaged sample or an adjacent section that shows comparable spatial features. Native samples, classic histological techniques (hematoxylin and eosin, toluidine), or immunohistochemistry staining (antibody staining) can be provided for this purpose.

The rest of the submission steps are identical to the MS proteomics “partial” submission process [13] (see Figs. S1–S10). An updated tutorial is available at http://www.proteomexchange.org/submission. Briefly, in step 4 (“Mapping Files”), the relationships between the different files can be captured. Each “RAW” file requires at least one “SEARCH” file. In addition, for MS imaging data, “RAW” files and “MS_IMAGE_DATA” files need to be connected. Step 5 is devoted to the annotation of biological and technical metadata. Information about species, tissues, and instrumentation is mandatory. In step 6, contact details for the principal investigator need to be provided. Step 7 is devoted to additional details. For instance, there it is possible to provide a PubMed identifier if the corresponding manuscript has already been published at submission time (as is the case in the example dataset). Step 8 (“Summary Screen”) provides an overview of all the files and file mappings, for the submitters to perform a final review before the actual file upload occurs. Step 9 is the final step where the files are uploaded to PRIDE. When the transfer is finished, the submitter will receive a confirmation e-mail. The actual time required to upload a dataset depends on the size of the dataset and the bandwidth available. In our example, it took approximately 1 h to prepare the data and collect the information for the submission, and the actual transfer of 830 MB data from Giessen to Cambridge, UK, took less than 5 min.

The data submission is then processed by the PRIDE team, and a “PXD identifier” is issued for each dataset. The submitter also receives by e-mail a username and password to allow private access to the data.

### Retrieve and display data from the repository

All submitted datasets are private by default. Each dataset becomes publicly available on acceptance or publication of the corresponding manuscript, or when the submitters tell PRIDE to do so. All public PX datasets (including those in PRIDE) are accessible via the portal ProteomeCentral (http://proteomecentral.proteomexchange.org/). There it is possible to search for PX datasets, independently from the receiving repository (Fig. S11). However, although the datasets are private, reviewers/editors need to go to PRIDE (http://www.ebi.ac.uk/pride) and log in using the username and password provided by the authors. Detailed information about how to access private datasets is provided in the electronic supplementary material.

The imzML data can be downloaded and viewed in freely available software tools such as MSiReader [16], Datacube Explorer [17], and OmniSpect [18]. Alternative commercial tools include Quantinetix [19] and MALDIVision [20]. An updated list of available tools is available at http://www.imzml.org.

**Fig. 2:**
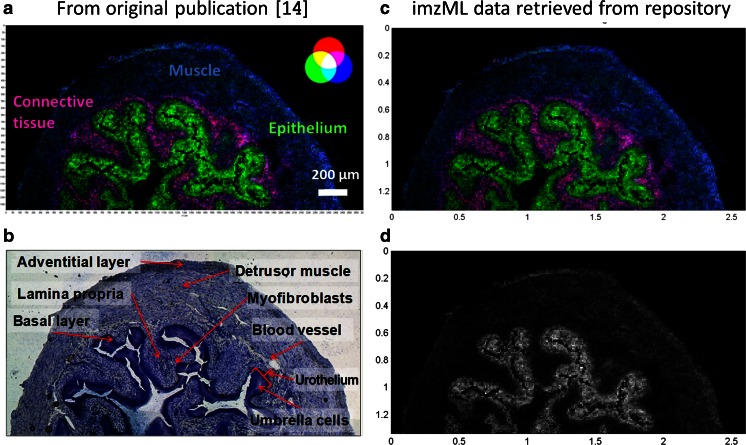
Submitted dataset as displayed in the original publication [14]: mouse urinary bladder (*A*, *B*). *A* overlay of selected ion images for *m*/*z* 741.5307 (*blue*, muscle tissue, sphingomyelin 34:1), *m*/*z* 798.5410 (*green*, urothelium, phosphatidylcholine 34:1), and *m*/*z* 743.5482 (*red*, lamina propria); bin size ∆*m*/*z* = 0.01, 10-μm pixel size, 260 × 134 pixels. *B* optical image of measured section stained with toluidine. Mass spectrometry images regenerated from the imzML dataset as retrieved from ProteomeXchange and displayed in the open source program MSiReader (*C*, *D*). *C* overlay of selected ion images corresponding to *A. D* selected ion image of *m*/*z* 798.5410 (phosphatidylcholine 34:1). (*A*, *B* modified from the original figure in Römpp et al. Copyright 2010 Wiley-VCH Verlag GmbH & Co. KGaA)

Panel C in Fig. 2 shows the imzML dataset as downloaded from PRIDE (accession number PXD001283) and displayed in the open source program MSiReader on a local computer (a screenshot that includes the user interface is shown in Fig. S12). The MS image shows features basically identical to those in the figure included in the original publication (Fig. 2, panel A). Alternatively, individual masses can be displayed as a grayscale image as shown for the example of *m*/*z* 798.5410 in Fig. 2, panel D (a corresponding MS image was included in the supporting online information of the original publication [14]). This MS image represents the raw data without any modifications such as interpolation or normalization. All necessary parameters to generate these MS images (bin width, color map, normalization, and interpolation options) are provided in the PX submission. This example demonstrates how MS imaging data used in publications can be reproduced with freely available and vendor-independent software. The reader can now access and search fully functional MS datasets, instead of evaluating the graphical representation of selected mass spectra and predefined MS images.

## Discussion and outlook

In this article we have provided an overview of the newly implemented process for submitting MS imaging datasets to PX via PRIDE. The procedure allows the evaluation of data during the manuscript reviewing process. In the MS proteomics field, there is a growing trend toward public data reuse which is triggering the assessment, reuse, comparative analysis, and extraction of new findings from already published data. As an example, one very prominent case of reuse of PX datasets occurred in the elaboration of one of the recently published “drafts” of the human proteome [21].

Likewise, submitted datasets of MS imaging data could be used as input for bioinformatics reanalysis by other groups. One particular type of data reuse, popular already in other disciplines, is to analyze data coming from a large number of publications/datasets in a combined way. This is a so-called meta-analysis study, which could also be applicable to MS imaging.

A possible next step would be to establish procedures for making possible PX “complete” submissions for MS imaging data. For this it would be necessary to agree on a standard procedure that can make online/direct data visualization possible and, if applicable, make possible the direct connection between the mass spectra and the compounds reported in a given study. The submitted exemplary dataset represents the first fully functional (independent of vendor software) MS imaging dataset that is publically available and that can be used freely for subsequent reprocessing and interpretation (the original publication [14] should be referenced in this case). For example, this dataset could be used as a reference and test dataset for developers of data processing software, i.e., to evaluate procedures for mass recalibration or spatial segmentation. We have been asked to provide data for such activities in the past, which triggered a discussion about how to share and exchange data and what the conditions for their reuse would be. Now interested parties can directly download the data and use the data as necessary. This submitted example also acts as a template for the submission of larger datasets, since the procedure and requirements are identical. Currently, there is no upper limit on the size of the submitted datasets. MS datasets with sizes up to a few terabytes have been submitted successfully to PX.

Our main objective here has been to show that a data repository is now available for MS imaging data. A relatively small dataset was chosen in order to facilitate data retrieval and processing, but the mechanism is also applicable for larger-scale data. We are not proposing this process as the only and default procedure for publication of MS imaging data in the immediate future, but rather see this as a demonstration that there are no longer any technical obstacles to a public repository for MS imaging data. The availability of such informatics tools represents an important step toward establishing MS imaging as a routine and reliable method in biomedical research. For instance, it may well open the route to fully integrate MS imaging within large-scale international research programs such as the Human Proteome Project.

The proposed approach allows public dissemination of MS imaging data based on existing public investment and infrastructure, avoiding significant extra costs for the development of a new, dedicated resource. The current setup allows the community to evaluate and test this submission process. We welcome any feedback since it could be used to improve and facilitate the submission process in the future.

## Electronic supplementary material

Below is the link to the electronic supplementary material.

ESM 1 (PDF 1.31 mb)
