# Supplementary material for: A public repository for mass spectrometry imaging data
Source: Anal Bioanal Chem. 2014 Dec 27;407(8):2027–33. doi: 10.1007/s00216-014-8357-8 (PMC4357645; doi:10.1007/s00216-014-8357-8)
Supplement: Supplementary file 1 — (PDF 1.31 mb) [file 216_2014_8357_MOESM1_ESM.pdf]

Analytical and Bioanalytical Chemistry

Electronic Supplementary Material

A public repository for mass spectrometry imaging data

Andreas Römpp, Rui Wang, Juan Pablo Albar, Andrea Urbani, Henning Hermjakob, Bernhard Spengler, Juan Antonio Vizcaíno

I. Information about the biological dataset used

Title:
Mass spectrometry imaging of phospholipids in mouse urinary bladder (imzML dataset)

Keywords:
Mass spectrometry imaging, tissue imaging, phospholipids, mouse urinary bladder, imzML

Project description:
The spatial distribution of phospholipids in a tissue section of mouse urinary bladder was analyzed by MALDI MS imaging at 10 micrometer pixel size with high mass resolution (using an LTQ Orbitrap mass spectrometer).

Sample preparation and experimental details:
All the experiments were performed with a AP-SMALDI imaging source. The laser was focused by a centrally bored objective lens to a diameter of 5 to 10 micrometer. Controller software and hardware for the scanning procedure were developed in-house. The imaging source was attached to a linear ion trap/Fourier transform orbital trapping MS (LTQ Orbitrap Discovery, Thermo Scientific GmbH, Bremen, Germany) with a mass resolving power of 30 000 at m/z 400 in positive-ion mode. This setup offers atmospheric pressure compatibility, MSn capability, and sub-ppm mass accuracy. A UV laser with a repetition rate of 60 Hz (LTB MNL-106, LTB, Berlin, Germany) was used for desorption/ionization. The mass range was m/z 100-1000 for measurements of phospholipids. Tissue sections (20 micrometer thickness) were coated with DHB (2,5-dihydroxybenzoic acid) matrix using a pneumatic sprayer. Assignments of lipids were confirmed by MS/MS analysis directly from tissue (isolation window dm/z 3). All images were generated with a bin width of dm/z 0.01. Details of sample preparation, data acquisition and data processing are described in: Römpp, A., S. Guenther, Y. Schober, O. Schulz, Z. Takats, W. Kummer and B. Spengler (2010). Angewandte Chemie International Edition 49(22): 3834-3838.

Data processing:
MS imaging data (Thermo RAW format) was converted to imzML (www.imzml.org) using the 'RAW to imzML' converter. For more information see: Schramm, T., A. Hester, I. Klinkert, J.-P. Both, R. M. A. Heeren, A. Brunelle, O. Laprevote, N. Desbenoit, M.-F. Robbe, M. Stoeckli, B. Spengler and A. Römpp (2012). Journal of Proteomics 75(16): 5106-5110. Selected ion images were generated using the software package MIRION developed at JLU Giessen. The imaging software imports raw data files as stored by the LTQ Orbitrap instrument software during image acquisition and couples this mass spectrometric information with additional scanning metadata, stored in separate data files by our ion source control program. This metadata includes the number of lines and columns of the image and the pixel size. The imaging software is able to create ion images from any of the detected mass-to-charge values with any selected mass window (bin width). A fast image browser of the MIRION software assists in selecting of images. In this work ion images of selected mass-to-charge values were created from the FT MS data set with a bin width of dm/z = 0.01. Up to three different ion images were overlayed in RGB images by the software to display different ion species in parallel. No other postprocessing steps such as interpolation or normalization to matrix signals were applied to the images.

II. Screenshots of all steps of submission process using the 'PX Submission Tool'

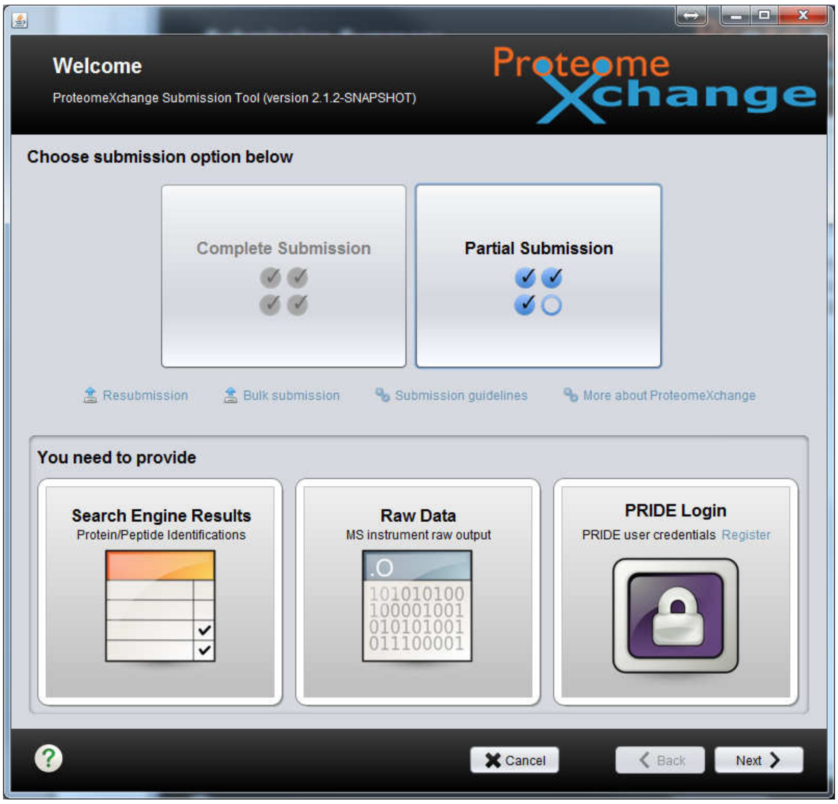

Fig. S1 Screenshot of the PX submission tool: Step 1

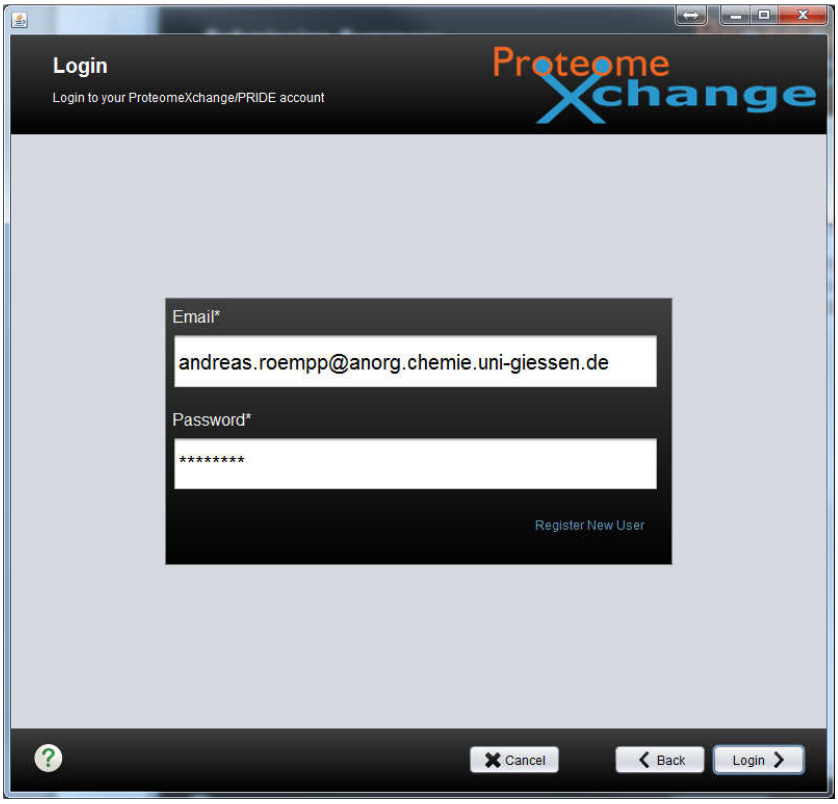

Fig. S2 Screenshot of the PX submission tool: Step 2

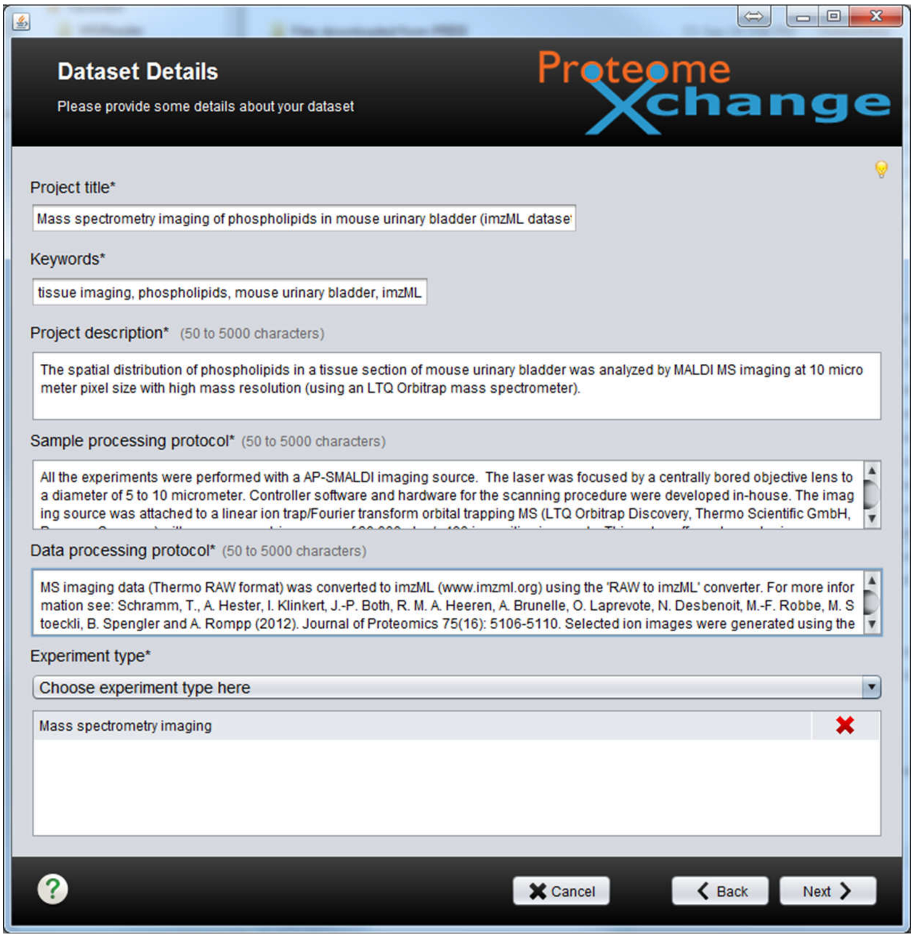

Fig. S3 Screenshot of the PX submission tool: Step 3

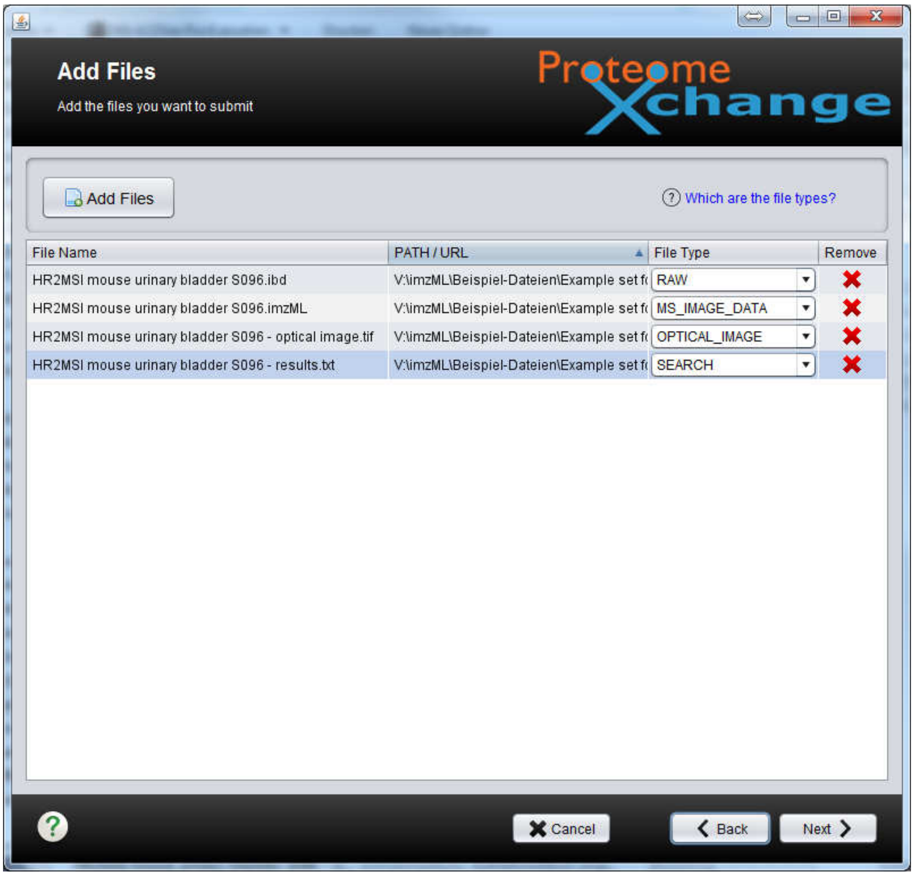

Fig. S4 Screenshot of the PX submission tool: Step 4

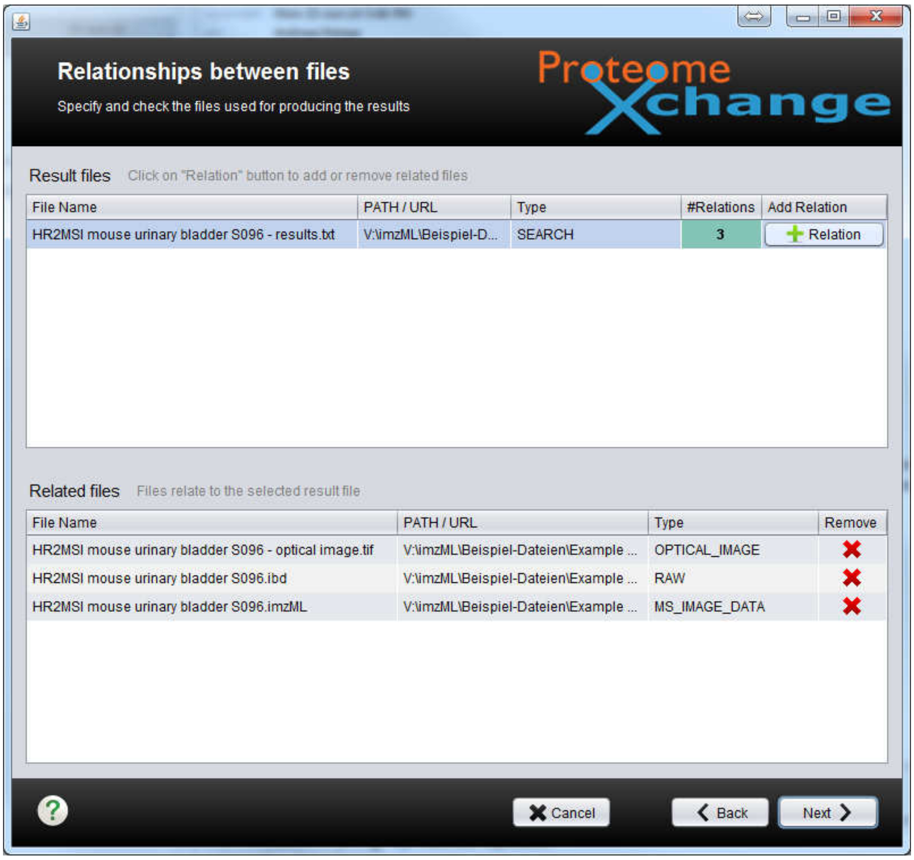

Fig. S5 Screenshot of the PX submission tool: Step 5

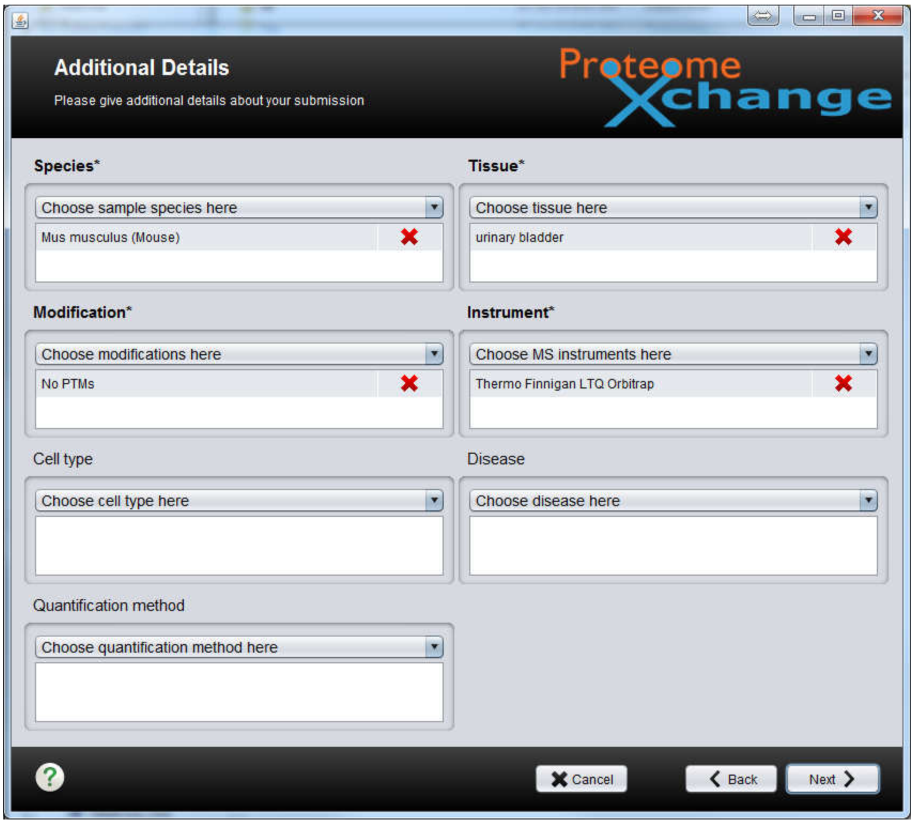

Fig. S6 Screenshot of the PX submission tool: Step 6

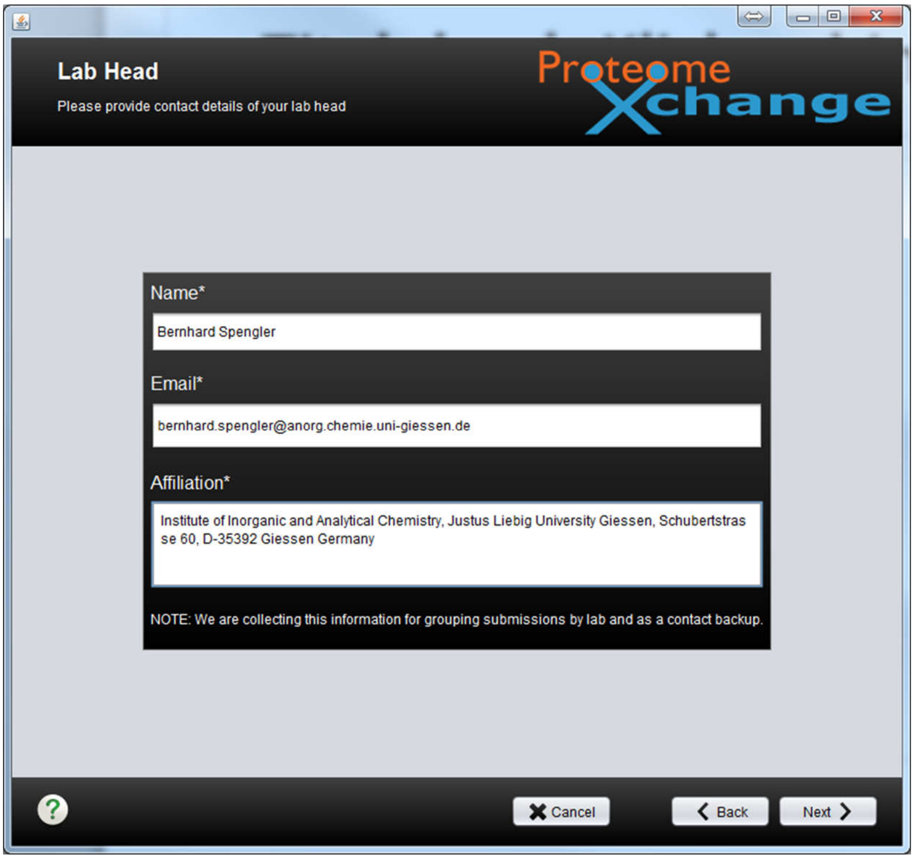

Fig. S7 Screenshot of the PX submission tool: Step 7

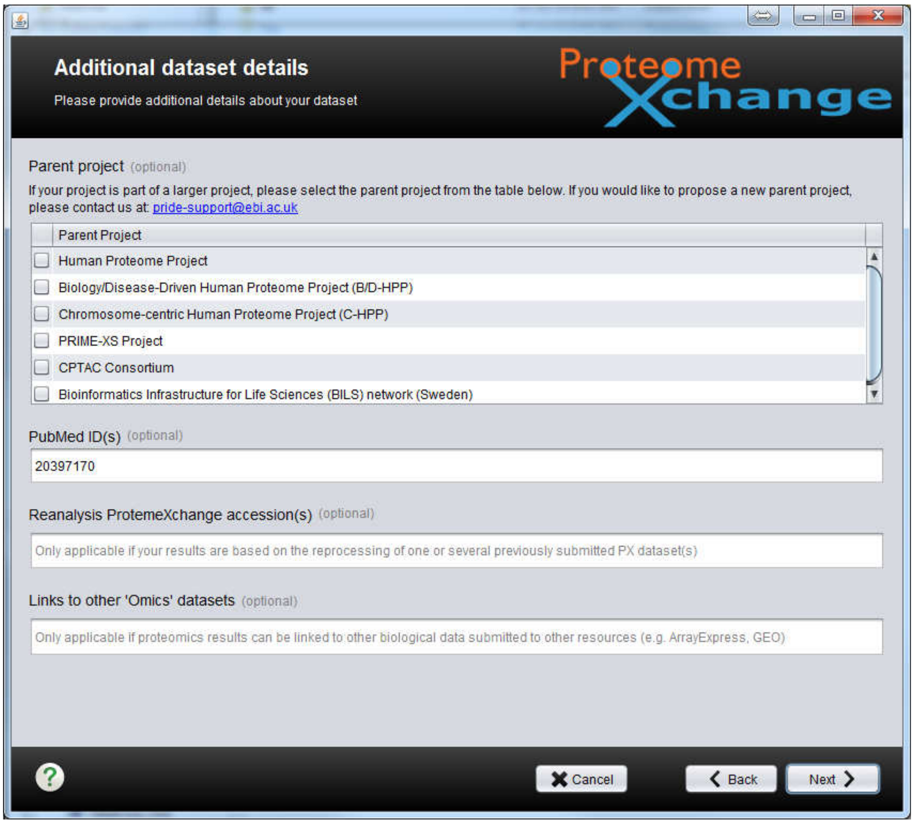

Fig. S8 Screenshot of the PX submission tool: Step 8

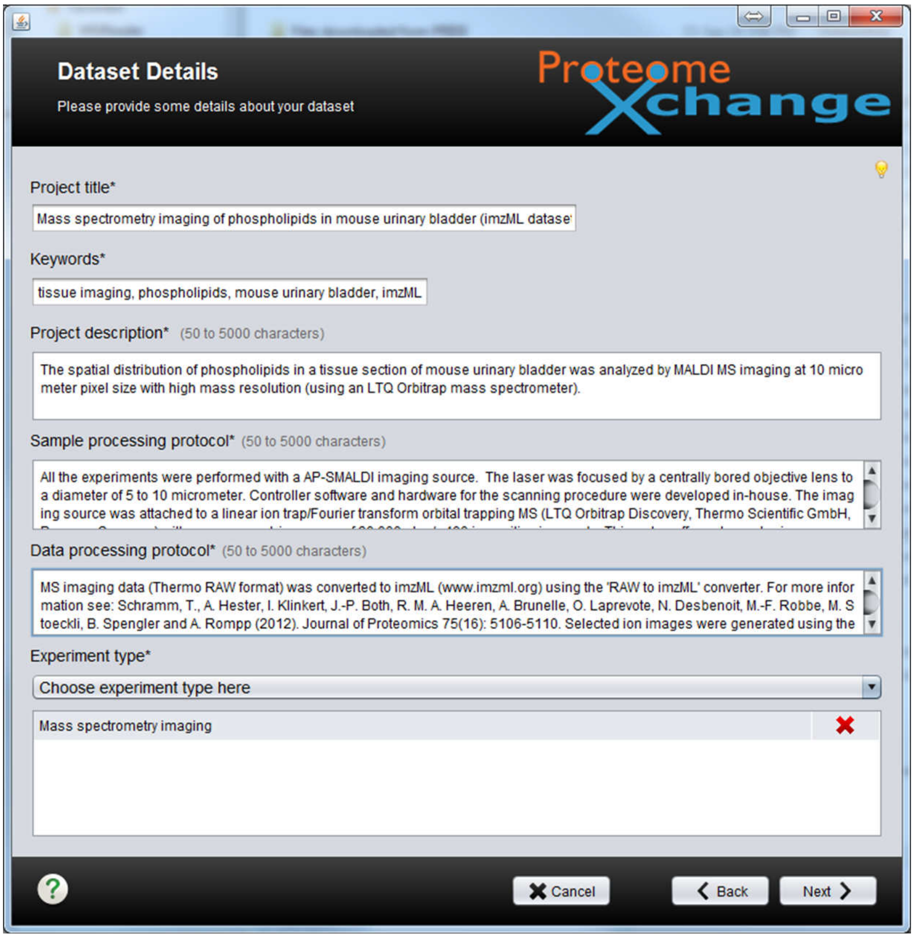

Fig. S9 Screenshot of the PX submission tool: Step 9

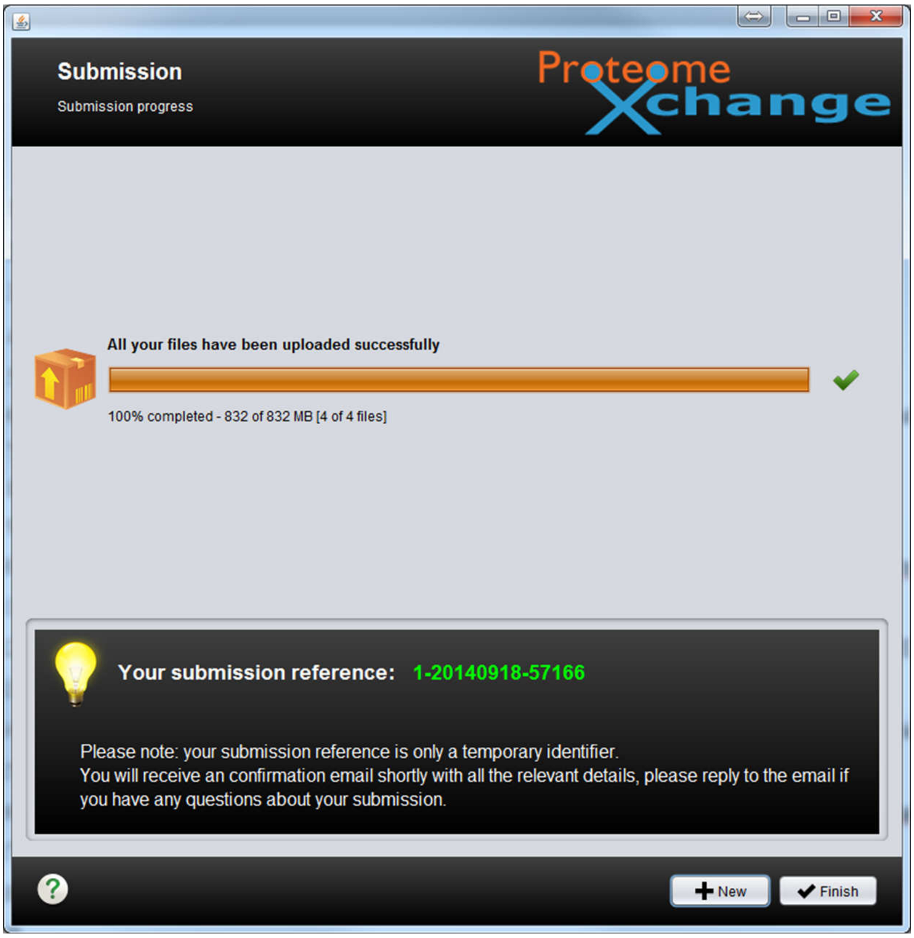

Fig. S10 Screenshot of the PX submission tool: Step 10

III. ‘SEARCH’ file included in the exemplary dataset

HR2MSI mouse urinary bladder S096 – results.txt (in csv format)

High resolution mass spectrometry imaging of mouse urinary bladder (project title);;;;
As published in Roempp A, Guenther S, Schober Y, Schulz O, Takats Z, Kummer W and Spengler B (2010) Angewandte Chemie International Edition 49 (22):3834-3838. (Figure 1, Figure S2);;;;
;;;;
Parameters used to generate MS images in publication:;;;;
Pixel size: 10 æm;;;;
Bin width ;;;;
Normalization: none;;;;
Interpolation: none;;;;
;;;;
;m/z;Compound;Bin width;
Figure 1A, RGB overlay;;;;
Red;743.5482;unknown;unknown;
Green;798.541;PC(34:1);[M+K]+;
Blue;741.5307;SM(34:1);[M+K]+;
Figure 1B, RGB overlay;;;;
Red;616.1767;Heme;M+;
Green;812.5566;PE(38:1);[M+K]+;
Blue;798.541;PC(34:1);[M+K]+;
Figure S2 (supporting information), grey scale images;;;;
;743.5482;Unknown;;
;741.5307;SM(34:1);[M+K]+;
;798.541;PC(34:1);[M+K]+;
;616.1767;Heme;M+;
;772.5253;PC(32:0);[M+K]+;

IV. Other screenshots

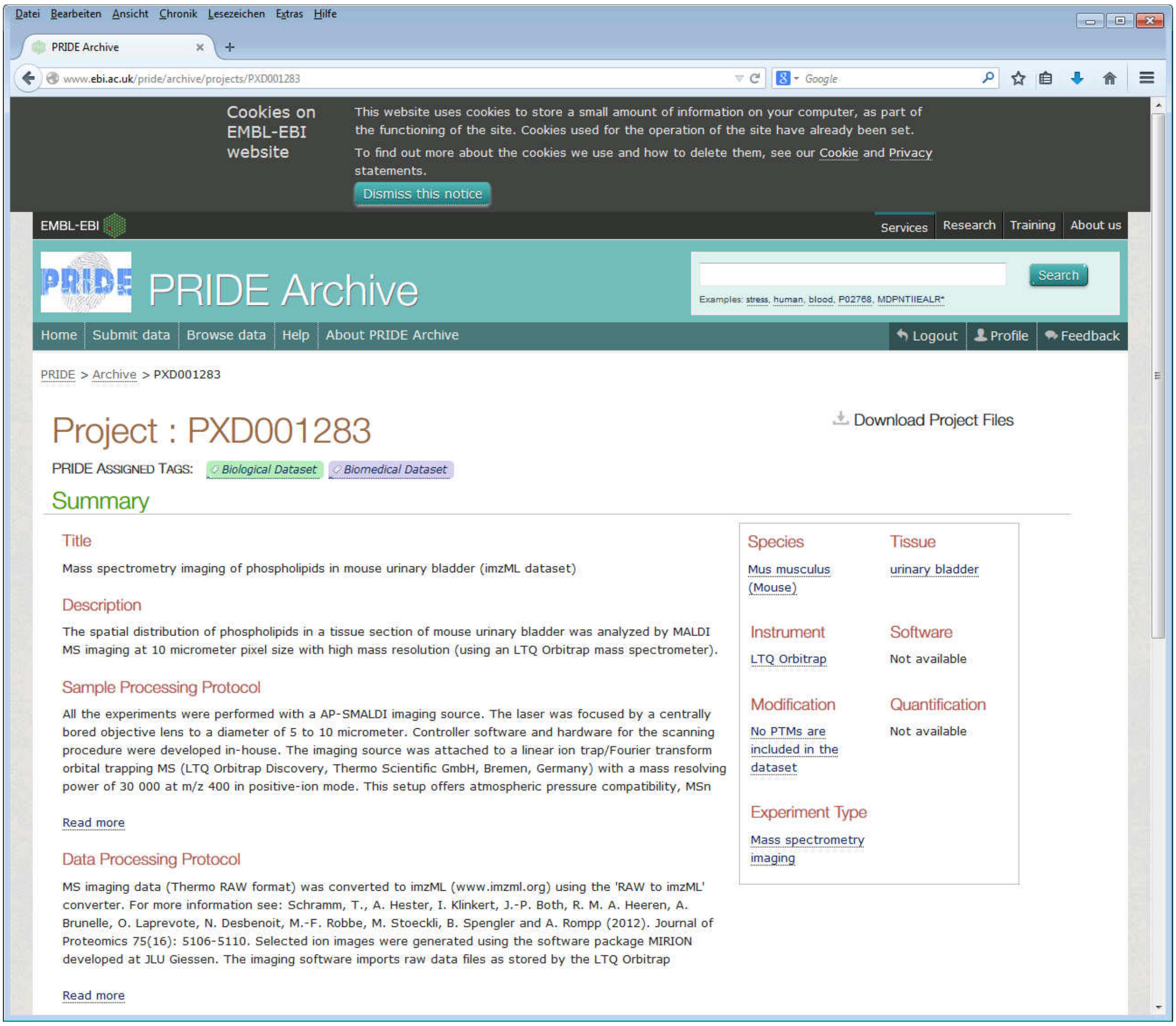

Fig. S11 Screenshot of the exemplary dataset in the PRIDE website

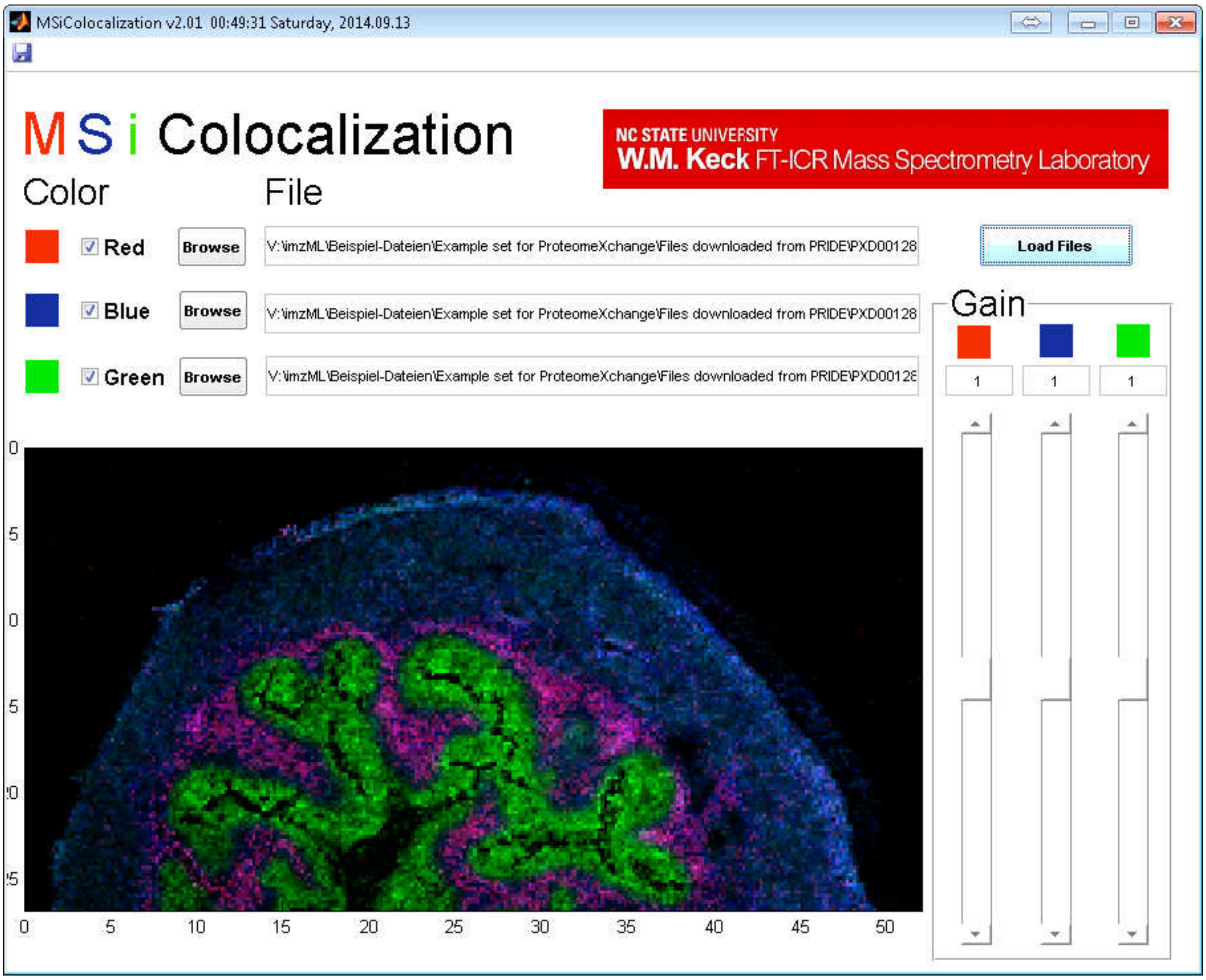

Fig. S12 Screenshot of MS imaging data displayed in MSiReader (data shown in Figure 2C)

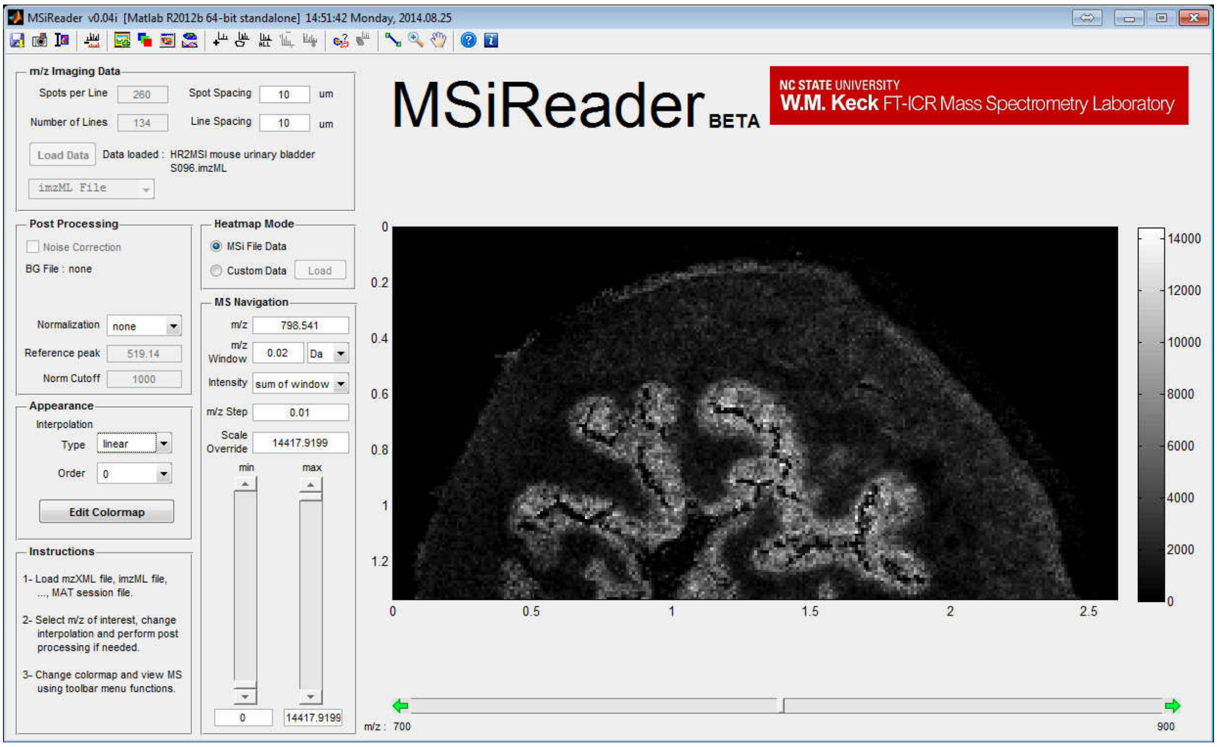

Fig. S13 Screenshot of MS imaging data displayed in MSiReader (data shown in Figure 2D)

V. Handling and modification after submission

How to access data privately in PRIDE

Private data set files can be accessed by journal editors/reviewers in two ways: *via* the PRIDE Archive web or *via* PRIDE Inspector.

(i) The PRIDE Archive web site (http://www.ebi.ac.uk/pride/archive). Reviewers need to log-in using the reviewer account (including username and password) provided by PRIDE. The details should be communicated by the authors, ideally in the main text.

(ii) In the PRIDE Inspector tool (which can be downloaded at http://code.google.com/p/pride-toolsuite/wiki/PRIDEInspector), select 'Review Project' and enter the account details (username and password). For MS imaging data, it is just possible to download all the files to a given directory since they are always 'partial' submissions).

How to modify an already submitted data set

A given data set can be modified while it remains private. This can be done through the 'Resubmission' option using the PX submission tool (available in Step 1). The whole data set needs to be submitted again.

How to make a data set public or add the corresponding published reference

By default, a dataset will be made publicly available after the related manuscript has been accepted, or when PRIDE staff is notified to do so by the original submitter. There are two ways to do it: (i) contacting the PRIDE team by e-mail, or (ii) using the PRIDE Archive website (http://www.ebi.ac.uk/pride/archive). To use the web option, the user will need to be logged in and click on the 'Publish' button located next to each unpublished dataset.

The corresponding reference associated with a given dataset can also be provided in both ways. It is encouraged that the final version of the reference is always provided. This could potentially be available quite some time after the actual acceptance of the manuscript.
